# Clustering of human prion protein and α-synuclein oligomers requires the prion protein N-terminus

**DOI:** 10.1038/s42003-020-1085-z

**Published:** 2020-07-09

**Authors:** Nadine S. Rösener, Lothar Gremer, Michael M. Wördehoff, Tatsiana Kupreichyk, Manuel Etzkorn, Philipp Neudecker, Wolfgang Hoyer

**Affiliations:** 1 Institute of Biological Information Processing (IBI-7) and JuStruct: Jülich Center for Structural Biology, Forschungszentrum Jülich, 52425 Jülich, Germany; 2 Institut für Physikalische Biologie, Heinrich-Heine-Universität Düsseldorf, 40204 Düsseldorf, Germany

**Keywords:** Biochemistry, Biophysics, Structural biology

## Abstract

The interaction of prion protein (PrP) and α-synuclein (αSyn) oligomers causes synaptic impairment that might trigger Parkinson’s disease and other synucleinopathies. Here, we report that αSyn oligomers (αSynO) cluster with human PrP (huPrP) into micron-sized condensates. Multivalency of αSyn within oligomers is required for condensation, since clustering with huPrP is not observed for monomeric αSyn. The stoichiometry of the heteroassemblies is well defined with an αSyn:huPrP molar ratio of about 1:1. The αSynO−huPrP interaction is of high affinity, signified by slow dissociation. The huPrP region responsible for condensation of αSynO, residues 95−111 in the intrinsically disordered N-terminus, corresponds to the region required for αSynO-mediated cognitive impairment. HuPrP, moreover, achieves co-clustering of αSynO and Alzheimer’s disease-associated amyloid-β oligomers, providing a case of a cross-interaction of two amyloidogenic proteins through an interlinking intrinsically disordered protein region. The results suggest that αSynO-mediated condensation of huPrP is involved in the pathogenesis of synucleinopathies.

## Abstract (web summary)

Nadine S. Rösener et al. show that α-synuclein oligomers can cluster with human prion protein (huPrP) into condensates. The intrinsically disordered N-terminus of huPrP is necessary for this high-affinity interaction, suggesting a link between cluster formation and toxicity.

## Introduction

Different supramolecular assembly types of amyloidogenic proteins have been implicated in neurodegenerative and non-neurological diseases. For example, amyloid fibrils of αSyn and amyloid-β (Aβ) are the main components of Lewy bodies and senile plaques, pathological inclusions found in Parkinson’s disease (PD), and Alzheimer’s disease (AD), respectively^1^. These aggregates can propagate and spread within the brain in a characteristic manner that is tightly linked to disease progression^2^. However, substantial evidence suggests that it is the smaller and more diffusible oligomeric assemblies that are triggering early pathogenesis^3–7^. Both αSyn oligomers (αSynO) and Aβ oligomers (AβO) can induce synaptic dysfunction and inhibit hippocampal long-term potentiation (LTP), an electrophysiological correlate of learning and memory^5,8,9^.

Apart from their role in PD, αSyn aggregates are the pathological hallmarks of dementia with Lewy bodies (DLB), multiple system atrophy, and other neurodegenerative diseases, collectively termed synucleinopathies^10^. Moreover, Lewy-body-like αSyn inclusions are found in most of the AD cases, signifying the pathological overlap between neurodegenerative diseases^11^. While αSyn is an intracellular protein, it is released in oligomeric form under stress conditions from neuronal cells and can spread to neighboring neurons^12^. Different toxic effects of αSynO have been reported, including impaired synaptic function, increased intracellular Ca^2+^ levels, increased production of reactive oxygen species, impaired protein degradation systems, and mitochondrial dysfunction^3^. αSynO might exert some of these effects directly by pore formation and membrane permeabilization, but recent evidence points to the importance of receptor-mediated mechanisms^9,12,13^. Two receptors shown to interact with αSynO are toll-like receptor 2 and PrP^9,13^.

PrP is a glycosylphosphatidylinositol (GPI)-anchored surface glycoprotein that is expressed at high levels in the brain. Misfolding of the cellular isoform of PrP (PrP^C^) to the scrapie isoform (PrP^Sc^) causes neurodegeneration in transmissible spongiform encephalopathies^14^. In addition to the scrapie isoform, cellular huPrP has also been implicated in neurodegeneration as it acts as a receptor for AβO^15^. Mature membrane-anchored cellular huPrP consists of amino acid residues 23−230, with an intrinsically disordered N-terminal half and a structured C-terminal half. AβO binds to the huPrP N-terminus^15–19^, which triggers a neurotoxic signaling cascade that may be responsible for early synaptic dysfunction in AD, involving metabotropic glutamate receptor 5 (mGluR5), Fyn kinase, and *N*-methyl-d-aspartate (NMDA) receptors^20,21^. Interestingly, the huPrP N-terminus also binds β-sheet-rich conformers of other proteins, suggesting that it plays a more general role in neurotoxicity and neuroprotection^22,23^.

Recently, Ferreira et al. reported that αSynO forms a complex with huPrP and induces phosphorylation of Fyn kinase via mGluR5^9^, the same mechanism as described for AβO toxicity^20,21^. Fyn kinase in turn mediates *N*-methyl-d-aspartate receptor phosphorylation, which leads to altered calcium homeostasis and synaptic deficits in αSyn transgenic mice^9^. To mediate αSynO signaling, the amino acid region 93−109 in the huPrP N-terminus is needed^9^, which is also involved in AβO binding^15–19^. Another study, on the other hand, questioned direct binding of αSynO to huPrP^24^.

Here, we investigated the interaction of αSynO with huPrP. In particular, we tested for higher-order heteroassociation, motivated by previous observations by us and others of the formation of large AβO:huPrP complexes^25,26^. Higher-order receptor−ligand complexes have important consequences for signaling^27,28^. We find that αSynO and huPrP in fact interact with high affinity to form micron-sized condensates of well-defined stoichiometry. The clustering of αSynO is driven by the same region in the intrinsically disordered N-terminus of huPrP that is responsible for mediating toxic effects of αSynO, suggesting a link between condensate formation and toxic signaling.

## Results

### High-molecular-weight complexes of αSynO and huPrP

**Fig. 1:**
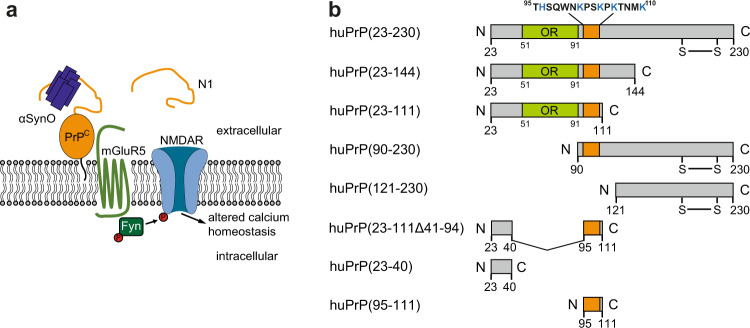
Interaction of αSynO with different huPrP variants. **a** Scheme of αSynO-PrP^C^ signaling. αSynO binds to membrane-anchored PrP^C^ and activates Fyn kinase via metabotropic glutamate receptor 5 (mGluR5), leading to phosphorylation of *N*-methyl-d-aspartate receptors (NMDAR) and finally to elevated intracellular calcium levels^9^. The naturally produced huPrP fragment N1 (residues 23−110/111) might prevent αSynO toxicity, analogous to its effect on AβO toxicity^17,29–31^. **b** Full-length huPrP(23−230) and seven fragments were investigated. OR octarepeat region. Orange, region needed for AβO binding^15–19^ which is almost the same region described to be necessary for αSynO binding^9^. huPrP(23−230), huPrP(90−230), and huPrP(121−230) contain a disulfide bond between Cys179 and Cys214.

**Fig. 2:**
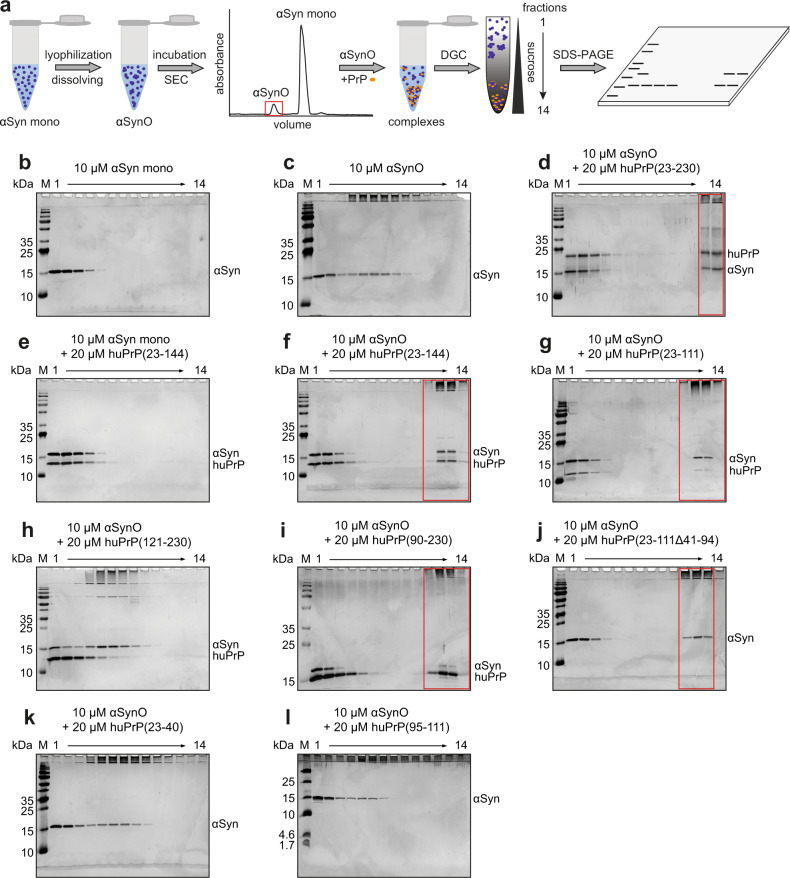
DGC analysis of heteroassembly formation by αSynO and different huPrP constructs. **a** Schematic of the assay. For preparation of αSynO, purified αSyn was dialyzed against water, lyophilized, redissolved in buffer, and incubated under agitation before separation by SEC. For further analysis, αSynO and huPrP were coincubated and separated by sucrose density gradient ultracentrifugation (DGC). Each DGC fraction was analyzed by SDS-PAGE. **b**–**l** Silver-stained Tris/Glycine (**b**–**k**) or Tris/Tricine (**l**) SDS-PAGE gels show the distributions of the applied proteins within the DGC gradients from left to right corresponding to the fractions from top to bottom of each gradient. Monomeric proteins are found in the top (left) fractions, oligomers in the middle fractions, and HMW species in the bottom (right) fractions. Lanes corresponding to HMW heteroassemblies are marked by red boxes. huPrP(23−111Δ41−94), huPrP(23−40), and huPrP(95−111) are not detected in the SDS-PAGE gels, probably due to their low molecular weights or to the high contents of basic amino acid residues.

αSynO was reported to bind to membrane-anchored PrP^C^ and activate Fyn kinase via metabotropic glutamate receptor 5 (mGluR5), leading to phosphorylation of *N*-methyl-d-aspartate receptors (NMDAR) and finally to elevated intracellular calcium levels (Fig. 1a)^9^. For studying the interaction of αSyn and huPrP, we investigated different huPrP fragments: full-length huPrP(23−230); the N-terminal fragments huPrP(23−144) and huPrP(23−111), the latter corresponding to the naturally produced and secreted huPrP fragment N1^17,29–31^; the C-terminal fragments huPrP(90−230) and huPrP(121−230); and three short fragments from the N-terminus, i.e., the 35 amino acid (aa) deletion fragment huPrP(23−111Δ41−94), the 18 aa peptide huPrP(23−40), and the 17 aa peptide huPrP(95−111) (Fig. 1b). The huPrP fragments were either of synthetic (the three short fragments from the N-terminus) or of recombinant origin (all other fragments), did not contain posttranslational modifications apart from the disulfide bond between Cys179 and Cys214, and were soluble in monomeric form as previously analyzed^26^. αSynO was prepared by lyophilization and agitation of αSyn according to a protocol based on Giehm et al.^32^ and Lorenzen et al.^33^. In short, purified αSyn was dialyzed against water, lyophilized, redissolved in buffer at a concentration of 12 mg ml^−1^ and incubated at 37 °C with shaking at 900 rpm for 3−5 h. Subsequently, the αSyn solution containing monomers as well as oligomers was separated by size exclusion chromatography (SEC) (Fig. 2a). Purified αSynO was coincubated with huPrP constructs to investigate their heteroassociation.

Sucrose density gradient ultracentrifugation (DGC) was used to analyze the size distribution of αSyn and huPrP assembly species. After ultracentrifugation, each DGC fraction was analyzed by silver-stained sodium dodecyl sulfate polyacrylamide gel electrophoresis (SDS-PAGE) (Fig. 2a). First, αSyn monomers (αSyn mono) and αSynO were separated by DGC for verification of their aggregation states (Fig. 2b, c). The given concentrations refer to monomer equivalents in the samples applied onto the gradient before centrifugation. The 14.5 kDa αSyn mono was found in the upper DGC fractions 1−4 (Fig. 2b). The αSynO sample showed a distribution of αSyn over fractions 1−9 (Fig. 2c), indicating the presence of oligomeric species in the denser fractions 4−9 as well as residual αSyn mono. The monomer content in αSynO samples accounted for 36 ± 4% of total αSyn according to reversed-phase (RP) HPLC (Supplementary Fig. 1). In the denser fractions 4−9, a substantial part of αSynO was observed in the stacking gel after SDS-PAGE and silver staining (Fig. 2c), probably due to the high stability of αSynO previously reported^34^.

Like αSyn mono, monomeric huPrP(23−230) elutes in the upper DGC fractions 1−4^26^. However, coincubation of αSynO with full-length huPrP(23−230) resulted in the formation of large species found in fractions 13−14 after DGC, which contained both αSyn and huPrP(23−230) (Fig. 2d), indicating heteroassociation of αSynO and huPrP(23−230) into high-molecular-weight (HMW) complexes. Simultaneously, the αSynO bands which were present in the absence of huPrP in fractions 4−9 disappeared upon incubation with huPrP(23−230). Remaining αSyn visible in fractions 1−3 likely represents residual αSyn mono in the αSynO preparation, whereas huPrP(23−230) visible in fractions 1−3 stems from an excess of huPrP(23−230) at the applied molar ratio (see the subsequent section). Similar observations were made when the N-terminal fragments huPrP(23−144) or huPrP(23–111) were used, demonstrating that the N-terminal half of huPrP is sufficient for heteroassociation with αSynO into large complexes (Fig. 2f, g). In contrast, when αSynO was replaced by αSyn mono, no large heteroassemblies were formed with huPrP(23−230), huPrP(23−144), or huPrP(23−111), showing that the oligomeric state of αSyn is a prerequisite for HMW complex formation (Fig. 2e, Supplementary Figs. 2b, 3b). Similarly, when the C-terminal construct huPrP(121−230) was coincubated with αSynO, HMW complex formation was not observed (Fig. 2h). Clustering of huPrP and αSynO hence depends on the N-terminus of huPrP. In the further analysis of the αSynO−huPrP interaction, below we mainly focus on the N-terminal fragment huPrP(23−144). However, we obtained very similar results for full-length huPrP(23−230) (Supplementary Fig. 2) and its N1 fragment huPrP(23−111) (Supplementary Fig. 3).

### Narrow range of αSyn:huPrP stoichiometry in heteroassemblies

**Fig. 3:**
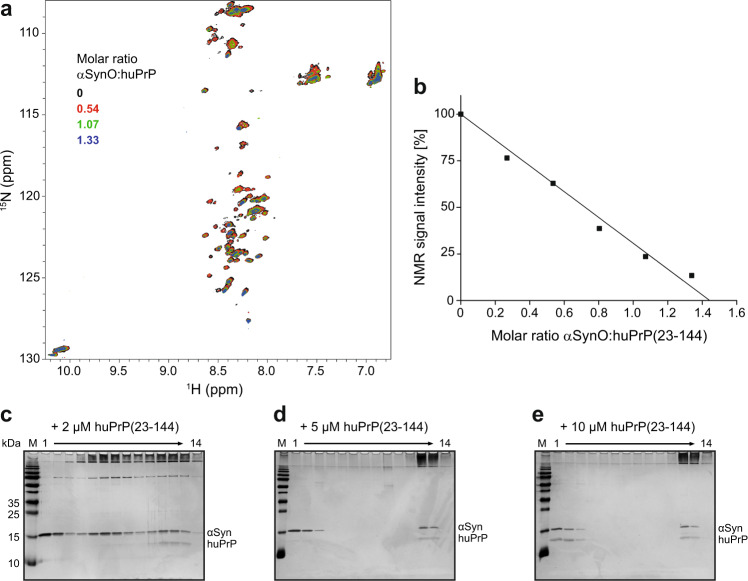
Determination of the αSyn:huPrP stoichiometry in heteroassemblies. **a** Addition of unlabeled αSynO to [U-^13^C,^15^N]-labeled huPrP(23−144) results in a global signal loss in [^1^H,^15^N] HSQC NMR. For a larger version of this panel, see Supplementary Fig. 6. **b** Total [^1^H,^15^N] HSQC NMR signal intensity in dependence of the αSynO:huPrP(23−144) molar ratio. The solid line represents a linear fit to the data. **c**–**e** Silver-stained SDS-PAGE gels after DGC of 10 µM αSynO coincubated with either **c** 2 µM, **d** 5 µM, or **e** 10 µM of huPrP(23−144).

Solution NMR spectra of [U-^13^C,^15^N]-labeled huPrP(23−144) show backbone amide resonances only in the random-coil region, in agreement with intrinsic disorder of this N-terminal fragment (Fig. 3a)^26^. Upon addition of unlabeled αSynO, these resonances show virtually no shift in resonance position but a marked decrease in intensity (Fig. 3a, b). This confirms that αSynO recruits [U-^13^C,^15^N]-huPrP(23-144) into large complexes, which are invisible in solution NMR due to their large size that results in a high rotational correlation time and hence very fast transverse relaxation, leaving only the monomeric fraction for detection. The NMR signal intensity decreases approximately linearly with the amount of αSynO added (Fig. 3b). This linear decrease in NMR signal intensity allows us to estimate the αSyn:huPrP stoichiometry in the HMW clusters. As illustrated in Fig. 3b, a linear fit to the NMR signal intensity decay data yields an αSyn:huPrP molar ratio of 1.45 ± 0.05 for complex formation in the case of an excess of huPrP (error represents the error of linear regression). Density gradient ultracentrifugation of samples containing 10 µM αSynO and different concentrations of huPrP(23−144) shows that free huPrP is visible (i.e., an excess of huPrP is present) above a total huPrP concentration of 5−10 µM (Fig. 3c–e). This is well in line with the ~1.5:1 αSyn:huPrP molar ratio determined by NMR, which predicts the emergence of free huPrP(23−144) in this DGC experiment at huPrP(23−144) concentrations above ~6.9 µM. Taking into account that the residual αSyn mono in the αSynO preparation (36 ± 3% of total αSyn, see Supplementary Fig. 1d) does not interact with huPrP, the NMR data yield an αSyn:huPrP molar ratio within the clusters of 0.92 ± 0.07 in the case of a huPrP excess.

In a DGC sample containing 2 µM huPrP(23−144) and 10 µM αSynO, an excess of uncomplexed αSynO is visible in fractions 4−9 (Fig. 3c). To evaluate to what extent the αSyn:huPrP stoichiometry within the HMW complexes differs between the cases of an excess of αSynO and an excess of huPrP, we aimed to determine the αSyn and huPrP contents of the heteroassemblies by HPLC. This method allows reliable quantitation of complex stoichiometries for the AβO−huPrP interaction^26,35^. For the αSynO−huPrP heteroassemblies, however, the method yields too low αSyn:huPrP stoichiometry values (Table 1), e.g., a ~0.55:1 αSyn:huPrP stoichiometry at an excess of huPrP, compared to the ~0.92:1 αSyn:huPrP stoichiometry determined by NMR and DGC. This likely stems from an underestimation of the αSyn content in the HMW fractions due to limited recovery in the quantitation by HPLC. Nevertheless, the method provides an estimate for the variability in αSyn:huPrP stoichiometry, with a twofold higher αSyn:huPrP molar ratio in the heteroassemblies at an excess of αSynO than at an excess of huPrP (Table 1). Taken together, the data demonstrate that the stoichiometry of the heteroassemblies falls into a narrow range, with a molar αSyn:huPrP ratio of about 1:1 in the presence of an excess of huPrP.

**Table 1:** αSyn:huPrP(23−144) ratios within the heteroassemblies after separation by sucrose DGC.

αSyn [µM]	huPrP(23−144) [µM]	αSyn:huPrP(23−144)^a^
10	2	0.96 ± 0.04
10	5	0.66 ± 0.03
10	10	0.55 ± 0.05
10	20	0.52 ± 0.07

^a^Experiments were done in replicates of *n* = 3, taken from distinct DGC samples. Errors represent SD. Protein contents in DGC fractions 11−14 were measured and quantified by RP-HPLC.

### αSynO and huPrP cluster into micron-sized particles

**Fig. 4:**
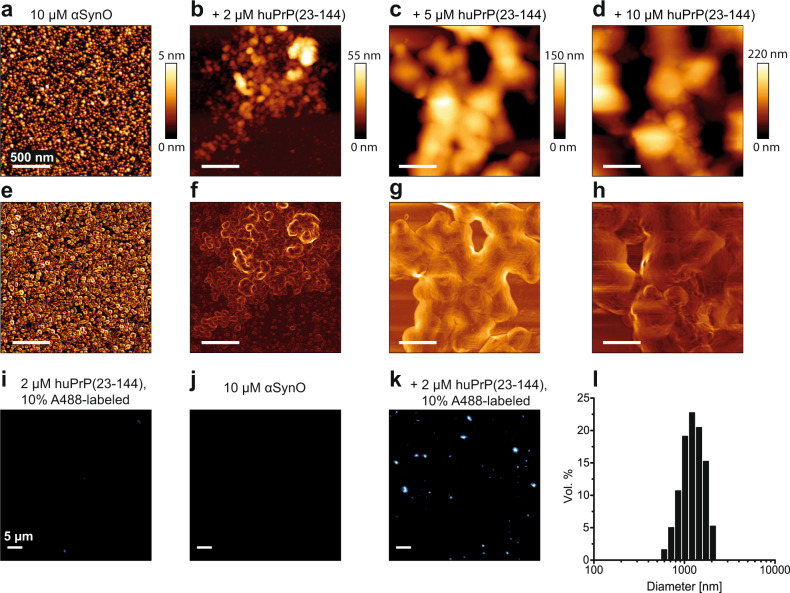
Analysis of αSynO and αSynO−huPrP(23−144) clusters. **a**–**h** AFM data are shown both as raw height images (**a**–**d**) and after edge detection using the Sobel operator for visualization of structural details (**e**–**h**). AFM scale bars represent 500 nm. **i**–**k** TIRFM shows the presence of clusters in a sample containing both 10 µM αSynO and 2 µM huPrP(23−144), 10% AlexaFluor488-labeled. **l** DLS measurement of heteroassemblies generated from 10 µM αSynO and 20 µM huPrP(23−144) and isolated by sucrose DGC.

αSyn can form a variety of oligomeric species in dependence of solution conditions^3,4^. When imaged by AFM after drying, αSynO prepared in this study are spherical objects 1.5−4 nm in height, with an apparent diameter of ~20 nm (Fig. 4a, e). This corresponds to a prevalent shape of αSynO observed in previous studies^3,4^. Heteroassemblies generated with 10 µM αSynO and 2 µM huPrP(23−144) consist of loose clusters with irregularly shaped spheres (Fig. 4b, f). These clusters have heights of up to 60 nm and measure up to 1.5 µm in width. In addition to the clusters there are still individual αSynO visible, in line with DGC showing uncomplexed αSynO under this condition (Fig. 3c). Keeping αSynO constant at 10 µM and increasing the huPrP(23−144) concentration to 5 µM (Fig. 4c, g) or to 10 µM (Fig. 4d, h) resulted in larger assemblies with heights of up to 250 nm and widths of several micrometers.

The heteroassemblies were also imaged by total internal reflection fluorescence microscopy (TIRFM). To visualize the interaction of αSynO with huPrP(23−144), 10% (mol/mol) of the huPrP(23−144) used in the TIRFM experiment was C-terminally labeled with an Alexa Fluor 488 dye. In the control sample containing only huPrP(23−144), barely any fluorescent particles are observed (Fig. 4i). The control sample containing only αSynO does not show any fluorescence, since αSyn was not fluorescently labeled (Fig. 4j). When the two components were mixed at concentrations of 10 µM αSynO and 2 µM huPrP(23−144), fluorescent condensates with sizes up to 2 µm formed (Fig. 4k), in agreement with the AFM data (Fig. 4b, f). Moreover, analysis of DGC-purified assemblies generated from 10 µM αSynO and 20 µM huPrP(23−144) by dynamic light scattering also confirmed the presence of large structures with diameters from 600 nm to 2 µm (Fig. 4l).

**Fig. 5:**
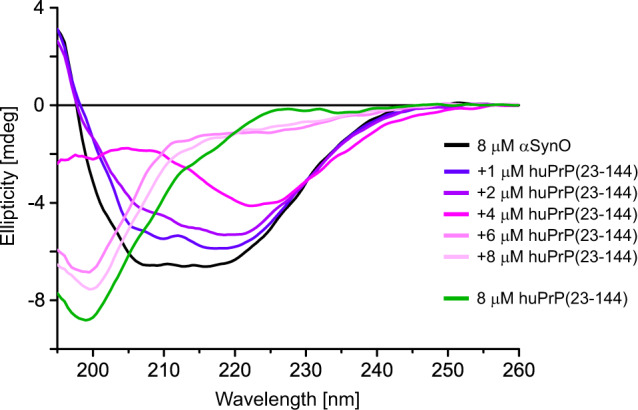
Absorption flattening upon cluster formation observed by far-UV CD spectroscopy. Spectra of free αSynO (black), free huPrP(23−144) (green), and αSynO titrated with huPrP(23−144).

Cluster formation was also investigated by circular dichroism (CD) spectroscopy. αSynO displays a broad minimum around 215 nm (Fig. 5), in agreement with previous data on this type of oligomer shown to be rich in β-structure^33^. In contrast, huPrP(23−144) exhibits a random coil spectrum with a minimum at 199 nm, reflecting its intrinsically disordered nature^26^. When increasing concentrations of huPrP(23−144) were added to 8 µM αSynO, the negative peak around 215 nm indicative of β-structure gradually lost intensity and shifted to higher wavelengths until it virtually vanished upon addition of 6 µM huPrP(23−144) (Fig. 5). At this molar ratio (αSyn:huPrP = 1.33), an excess of huPrP(23−144) is present as inferred from the NMR and DGC data, in agreement with the appearance of a random coil band with a minimum at 200 nm in CD. The loss of the β-structure band of αSynO upon cluster formation can be explained with absorption flattening, i.e., loss of absorbance due to the condensation of chromophores into colloids^36^. Absorption flattening is particularly prominent in CD spectroscopy and occurs when colloids increase in size from the nanometer to the micrometer scale^37,38^. Since absorption flattening is not uniform across the wavelength range, CD spectra do not only reduce in intensity but are also distorted, explaining the gradual shift of the αSynO β-structure signal. Due to the convolution with differential absorption flattening, potential secondary structure changes upon cluster formation cannot be deduced from the CD data.

### High affinity of the αSynO−huPrP interaction

**Fig. 6:**
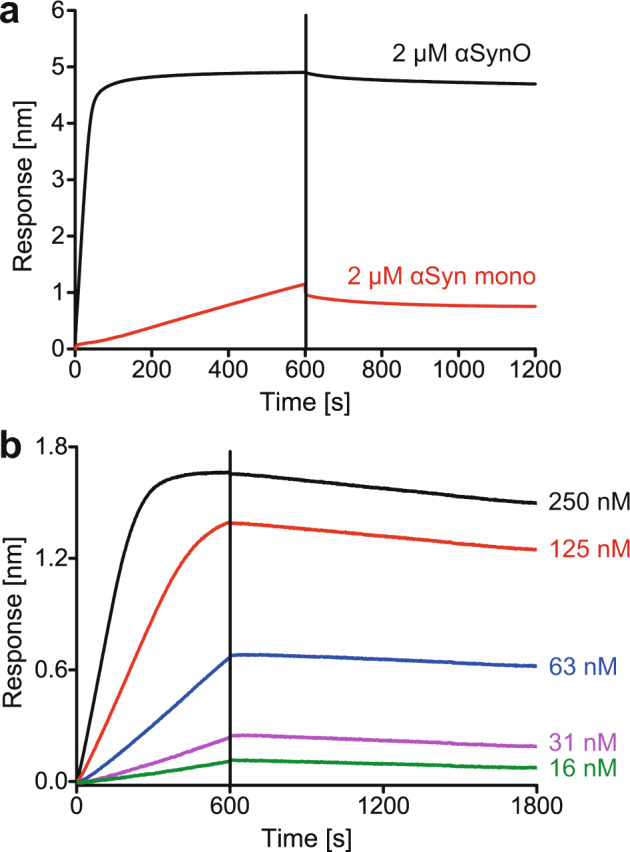
αSynO binds to huPrP(23−144) with high affinity. BLI sensorgrams of αSyn mono−huPrP(23−144) (**a**) and αSynO−huPrP(23−144) (**a**, **b**) interactions. Biotinylated huPrP(23−144) (carrying a C-terminal Cys for biotinylation) was coupled to streptavidin biosensors and αSyn was used as analyte. **a** Comparison of binding of αSyn mono and αSynO. **b** Concentration-dependent binding of αSynO. Association occurred from 0 to 600 s (**a**, **b**), dissociation from 600 to 1200 s (**a**) or 600 to 1800 s (**b**).

Biolayer interferometry (BLI) was performed to investigate the affinity of the αSynO−huPrP interaction. HuPrP(23−144), which was biotinylated through a C-terminal cysteine, was attached to streptavidin biosensors and its binding to 2 µM of either αSyn mono or αSynO was monitored (Fig. 6a). The BLI response to αSynO greatly exceeded that to αSyn mono, in line with the DGC data showing that the oligomeric state of αSynO is a prerequisite for cluster formation (Fig. 2e, f). A dilution series from 250 to 15.6 nM αSynO showed concentration-dependent binding to huPrP(23−144) (Fig. 6b). The very slow dissociation observed in BLI demonstrates that the αSynO−huPrP heteroassociates possess a high kinetic stability. Due to the lack of an established molecular interaction model applicable to coclustering of αSynO and huPrP, curve fitting was not applied to the BLI data. Nevertheless, BLI showed that the αSynO−huPrP interaction is of high affinity.

### The region 95−111 of huPrP is required for αSynO clustering

For a more precise localization of the αSynO-binding site within the huPrP N-terminus, we performed DGC with four further huPrP fragments, huPrP(90−230), huPrP(23−111Δ41−94), huPrP(23−40), and huPrP(95−111) (Fig. 1b). These fragments all lack the octarepeat region (residues 51−91) but contain either the N-terminal (residues 23−27), the C-terminal (residues ~95−110), or both of the basic sequence segments that are responsible for AβO binding^15–19^. Addition of 20 µM of either huPrP(90−230) or huPrP(23−111Δ41−94) to 10 µM αSynO resulted in the formation of large aggregates visible in DGC fractions 11−13 (Fig. 2i, j). In the case of huPrP(23−111Δ41−94), only αSyn can be observed in SDS-PAGE gels as the detectability of this huPrP fragment is limited, probably due to its low molecular weight or the high content of basic amino acid residues. However, RP-HPLC measurements confirmed the presence of huPrP(23−111Δ41−94) within the HMW fractions (Supplementary Fig. 1b). Coincubation of huPrP(23−40) and αSynO (Fig. 2k) resulted in the same distribution of αSyn over DGC fractions as that of αSynO alone (Fig. 2c), demonstrating that huPrP(23−40) is not able to cluster αSynO. Like huPrP(23−111Δ41−94), huPrP(23−40) could not be detected by silver-stained SDS-PAGE. Combining the results, all huPrP constructs that were able to cluster with αSynO contain amino acids 95−111, other specific huPrP regions were not obligatory. Interestingly, this region correlates well with the region comprising residues 93−109 which is required for aSynO-mediated inhibition of LTP^9^. We tested if huPrP(95−111) alone is sufficient for αSynO clustering. Coincubation with huPrP(95−111) did not shift the distribution of αSynO to higher MW; like the other short, basic peptides huPrP(95−111) could not be detected by silver-stained SDS-PAGE (Fig. 2l). The 17 aa peptide huPrP(95−111) does therefore not achieve clustering of αSynO on its own, but requires additional polypeptide segments which can stem from huPrP regions both N-terminal and C-terminal of residues 95−111.

### αSynO and AβO cocluster with huPrP

**Fig. 7:**
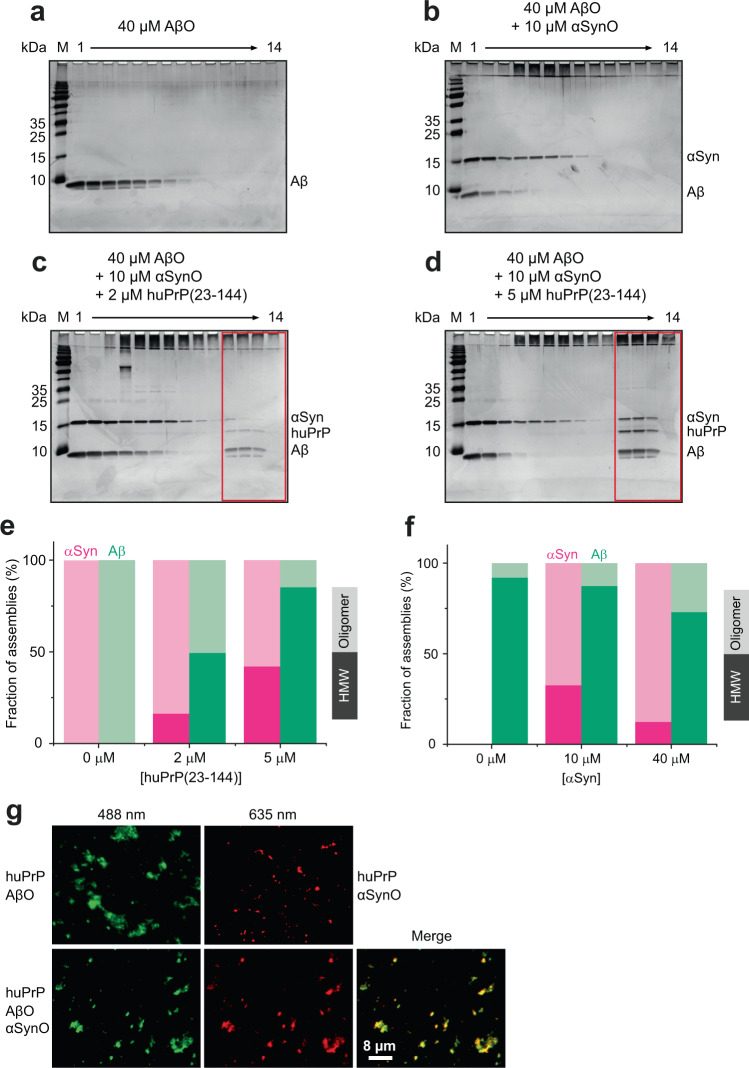
αSynO and AβO cocluster with huPrP(23−144). **a**–**d** Silver-stained SDS-PAGE gels after DGC of 40 µM AβO alone (**a**) and mixtures of 40 µM AβO and 10 µM αSynO containing either **b** no, **c** 2 µM, or **d** 5 µM huPrP(23−144). Coclustering of all proteins as heteroassemblies is detectable in DGC fractions 11−14 (red boxes). **e**, **f** Quantitative analysis by RP-HPLC of the distribution of assemblies into oligomer fractions (4−9) vs. HMW fractions (10−14). In (**e**), different amounts of huPrP(23−144) were added to a mixture of 40 µM AβO and 10 µM αSynO, corresponding to the gel images in (**b**–**d**). In (**f**), different amounts of αSynO were added to a mixture of 4 µM huPrP(23−144) and 40 µM AβO, corresponding to the gel images in Supplementary Fig. 4. **g** TIRFM of AβO (top left, 40 µM, 10% FITC-labeled), αSynO (top right, 10 µM, 10% ATTO633-labeled), and a mixture of both (bottom row, 40 µM AβO, 10% FITC-labeled, and 10 µM αSynO, 10% ATTO633-labeled), all in the presence of 2 µM huPrP(23−144). Excitation wavelengths were 488 nm for AβO (left) and 635 nm for αSynO (middle), emission wavelengths were 525 nm for AβO and 705 nm for αSynO. A merged image of the AβO and αSynO fluorescence channels is shown on the right.

Heteroassociation of αSynO and huPrP replicates many features previously observed for the AβO−huPrP interaction^26^. In both cases, nanometer-sized oligomers cluster into micron-sized condensates upon interaction with huPrP. In both cases, the stoichiometry in the clusters is well-defined, i.e., it shows only a limited dependence on the total concentrations of the components. At an excess of huPrP, the αSyn:huPrP ratio in the clusters is approximately 1:1 compared to an Aβ:huPrP ratio of 4:1^26^, indicating that per huPrP molecule ~4-fold more Aβ than αSyn molecules are bound. We investigated if huPrP preferentially triggers condensation of either αSynO or AβO. 40 µM of AβO, prepared using Aβ(1−42), shows an Aβ distribution covering DGC fractions 1−8 but no HMW assemblies (i.e. fibrils) in fractions 11−14 (Fig. 7a). To test for a preference of huPrP for heteroassociation with αSynO or AβO, we mixed huPrP(23−144) with 10 µM αSynO and 40 µM AβO, accounting for the ~4-fold higher binding capacity of huPrP for AβO than for αSynO. In the absence of huPrP, the mixture of 10 µM αSynO and 40 µM AβO did not show any Aβ or αSyn in HMW fractions (Fig. 7b). Upon addition of huPrP(23−144), all three proteins coclustered as heteroassemblies that are detectable in DGC fractions 11−14 (Fig. 7c, d). Quantitative analysis of the oligomer fractions (4−9) and HMW fractions (10−14) by RP-HPLC (Supplementary Fig. 1c, e) revealed that condensation of αSynO and AβO occurred in parallel, but that a larger fraction of AβO was shifted to HMW fractions as compared to αSynO (Fig. 7e). This result was reproduced also for huPrP(23−230) (Supplementary Fig. 2i, j) and huPrP(23−111) (Supplementary Fig. 3j, k). This indicates that huPrP has a higher affinity for AβO than for αSynO. The experiment was repeated using constant concentrations of huPrP(23−144) (4 µM) and AβO (40 µM), but variable concentrations of αSynO (0, 10, or 40 µM). Increasing concentrations of αSynO progressively displace AβO from the huPrP-induced clusters, which is evident from the reduced fraction of AβO in HMW fractions at higher αSynO concentration (Fig. 7f, Supplementary Fig. 4). This demonstrates that αSynO and AβO compete for huPrP, which is in line with the finding that the huPrP region comprising residues 95−111 is required for both αSynO and AβO binding. Again, a larger fraction of AβO was shifted to HMW fractions as compared to αSynO, both when αSynO and AβO were present at concentrations corresponding to similar huPrP binding capacity (10 µM αSynO and 40 µM AβO) and at equimolar concentration (40 µM αSynO and 40 µM AβO), confirming that huPrP has a higher affinity for AβO than for αSynO. The reduced fraction of αSynO in HMW fractions at 40 µM αSynO as compared to 10 µM αSynO is due to the fact that the amount of huPrP(23−144) is limited in this experiment. Only a minor fraction of the extra αSynO in the 40 µM αSynO sample displaces AβO from huPrP(23−144), the majority remains in the oligomer fraction. In addition, TIRFM was employed to confirm that αSynO and AβO cocluster in mixed condensates. Together with huPrP(23−144), fluorescein isothiocyanate (FITC)-labeled AβO and ATTO633-labeled αSynO indeed colocalized in micron-sized particles (Fig. 7g). The particles seen in the TIRFM images fluoresce upon 488 nm excitation (AβO), as well as upon 635 nm excitation (αSynO). Moreover, Förster resonance energy transfer was observed between 488 nm-excited AβO (donor) and αSynO (acceptor), as ~16% fluorescence intensity (compared to the donor fluorescence) was detected in the αSynO fluorescence channel upon excitation of AβO at 488 nm (Supplementary Fig. 5). This further highlights a close proximity on the nanometer scale of AβO and αSynO in mixed condensates.

## Discussion

**Fig. 8:**
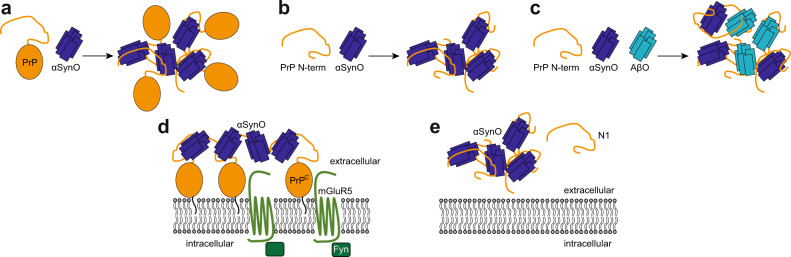
Scheme of αSynO−huPrP cluster formation. **a**–**c** Cluster formation observed in this study. HuPrP clusters with αSynO through the intrinsically disordered huPrP N-terminus (**a**). The huPrP N-terminus is sufficient for αSynO condensation (**b**). αSynO and AβO cocluster with huPrP (**c**). **d**–**e** Potential cluster formation of PrP^C^ or its N-terminal fragment N1 in vivo. PrP^C^ cluster formation may affect neurotoxic signaling of αSynO by promoting assembly of higher-order signaling complexes (**d**). Removal of neurotoxic amyloid oligomers by cluster formation may contribute to the neuroprotective activity of N1 (**e**).

The interaction of αSynO with the membrane surface receptor PrP^C^ was recently shown to activate neurotoxic signaling through mGluR5, Fyn kinase, and NMDA receptors, resulting in altered calcium homeostasis and synaptic impairment in mice (Fig. 1a)^9^. Using soluble huPrP constructs, we find that αSynO and huPrP interact with high affinity to cocluster into micron-sized condensates (Fig. 8a–c). The huPrP region required for αSynO-induced synaptic impairment (residues 93−109)^9^ also drives αSynO condensation (residues 95−111), suggesting that cluster formation may be involved in neurotoxic signaling (Fig. 8d). The in vitro experiments described here focus on the biophysical characterization of αSynO−huPrP condensate formation and do not prove a causal link between condensation and the pathophysiological activity of the αSynO−huPrP interaction. However, in support of a role of huPrP condensation in signaling, clustering of PrP^C^ was previously found to activate Fyn kinase^39,40^. Clustering of membrane-bound PrP^C^ is promoted by the enrichment of GPI-anchored proteins in submicron domains at the cell surface^41^. The assembly of receptors into higher-order signaling machines is prevalent in signaling cascades and enables specific modes of signal transduction^28^. Receptor clustering is frequently driven by interactions of intrinsically disordered segments of cell surface receptors with multivalent ligands^27^. In line with this, condensate formation of huPrP and αSynO involves an intrinsically disordered region of huPrP and requires an oligomeric, hence multivalent, state of αSyn. Due to its multivalency, a single αSynO may be sufficient to cluster multiple PrP^C^. At the same time, the membrane anchorage of PrP^C^ is unlikely to prohibit huPrP from cross-linking multiple αSynO, since PrP^C^ is GPI-anchored at its C-terminus and the intrinsically disordered N-terminus has sufficient conformational freedom to cross-link αSynO.

Apart from membrane-bound PrP^C^, the secreted, soluble N-terminal huPrP fragment N1 (Fig. 1a), which comprises amino acids 23−110/111, contains the region responsible for αSynO condensation and may therefore also cocluster with αSynO (Fig. 8e). Removal of neurotoxic amyloid oligomers by cocluster formation in the extracellular space would inhibit neurotoxic signaling and could contribute to the neuroprotective effect observed for this naturally produced huPrP fragment^17,26,30,31^.

The αSyn:huPrP stoichiometry in the heteroassemblies is approximately 1:1. Importantly, this does not mean that a defined 1:1 complex between one αSyn molecule and one huPrP molecule is formed. This is evident, for example, from the variability of the αSyn:huPrP ratio within the heteroassemblies, which is twice as high at an excess of αSynO than at an excess of huPrP (Table 1). Cluster formation of αSynO and huPrP instead reflects cross-linking of multivalent binding partners. Multivalency is inherent to αSynO due to its oligomeric nature. On the huPrP side, multivalency is probably supported by the intrinsic disorder of the N-terminus, in line with the critical role of intrinsically disordered regions in biomolecular condensation^42^. The precise contributions of side chain and backbone of specific amino acids in huPrP to cluster formation are not disclosed by the present data. However, the different huPrP fragments studied here lead to the conclusion that the region 95−111 is essential for cluster formation and requires support from an additional, not uniquely defined, polypeptide segment.

While the αSynO−huPrP interaction replicates many features previously observed for the AβO−huPrP interaction^26^, a difference is that per huPrP molecule ~4-fold more Aβ than αSyn molecules are bound. This factor corresponds well to the difference in sequence length between Aβ (42 aa) and αSyn (140 aa), suggesting that protein size co-determines the stoichiometry. With regard to the binding site, both αSynO and AβO interact with the intrinsically disordered huPrP N-terminus. Both require the huPrP region 95−111 for cluster formation. AβO condensation additionally depends on the huPrP region 23−27^26^. Similarly, αSynO condensation is not achieved by huPrP(95−111) alone but requires an additional polypeptide segment, which can be contributed from the very N-terminus of huPrP as in the case of AβO condensation. In contrast to AβO condensation, however, the additional polypeptide can also stem from C-terminal regions of huPrP, as is evident from the efficient cluster formation of αSynO and huPrP(90−230). HuPrP interacts with both αSynO and AβO with high affinity, yet it shows some preference for AβO (Fig. 7e, f). We find that huPrP can form mixed condensates containing both αSynO and AβO (Fig. 8c). This suggests that αSynO and AβO may reinforce each other’s neurotoxic signaling by employing huPrP as common signaling hub. Such a concerted activity of αSynO and AβO could contribute to the observed overlap of αSyn and Aβ pathologies^11^. In this context, it is interesting to note that recent studies found that interactors of the huPrP N-terminus differ between healthy and pathophysiological conditions^23^, and that AβO are not the only biomolecules triggering huPrP condensation in AD brain^25^. Our results show that αSynO is a further species forming condensates with huPrP and suggest a link between condensate formation and toxic signaling.

## Methods

### Purification of huPrP

Purification of recombinant huPrP fragments (huPrP(23−230), huPrP(23−144), huPrP(90−230), and huPrP(121−230)) was performed as described previously^26^. For preparation of huPrP(23−144)-Cys, its gene was cloned into pET 302/NT-His, expressed in *Escherichia coli* BL21 DE3 and purified under same conditions as described previously for huPrP(23−144)^26^. To ensure that monomeric and reduced huPrP(23−144)-Cys is obtained for subsequent maleimide labeling, the sample was reduced with 25 mM Tris(2-carboxyethyl)phosphin (TCEP) before application to final purification by RP-HPLC.

For preparation of huPrP(23−144)-Cys-biotinyl, a tenfold molar excess of freshly dissolved biotinyl-PEG2-maleimide (Bachem) in 200 mM HEPES/NaOH buffer pH 7.5 was added to lyophilized huPrP(23−144)-Cys. After incubation for 2 h at 25 °C at 600 rpm shaking huPrP(23−144)-Cys-biotinyl was purified from the reaction mixture by RP-HPLC on a Zorbax 300 SB-C8, 9.4 mm × 250 mm column (Agilent) using a 20-min gradient from 16 to 40% acetonitrile, 0.1% trifluoroacetic acid (TFA) in Milli-Q water at 4 ml min^−1^ flow rate and 80 °C column temperature. Eluted huPrP(23−144)-Cys-biotinyl was collected, aliquoted, and lyophilized.

AlexaFluor488 labeling of huPrP(23−144)-Cys was performed by adding a tenfold molar excess of *N*,*N*-dimethylformamide predissolved AlexaFluor488 C5 maleimide (Thermo Fisher) in 200 mM HEPES/NaOH buffer pH 7.5 to lyophilized huPrP(23−144)-Cys. After incubation for 2 h at 25 °C at 600 rpm shaking huPrP(23−144)-Cys-AlexaFluor488 was purified from the reaction mixture by RP-HPLC as described above.

The construct huPrP(23−111) was cloned by In-Fusion Cloning using the In-Fusion EcoDry Cloning Kit (Takara Bio USA Inc.). As template, huPrP(23−144) in a pET 302/NT-His vector was used. The required 15-bp overhangs were created by appropriate primers so that the regions 112−144 were not amplified in PCR. After successful In-Fusion reactions, these sequences were deleted. *E. coli* BL21 (DE3) was transformed with the plasmid and grown in 2YT medium at 37 °C and 110 rpm shaking. At an OD_600_ of 0.5, recombinant protein expression was induced by adding 1 mM isopropyl 1-thio-*β*-d-galactopyranoside, and after further 3 h, the growth temperature was lowered to 25 °C. Cells were harvested the next day and resuspended in 3 ml of digestion buffer (1× phosphate buffered saline, 20 mM MgCl_2_, DNAse I containing protease inhibitor mixture (Complete EDTA-free, Roche Applied Science, one tablet/50 ml)) per gram of cells and stored at −20 °C.

The volume of the *E. coli* cells containing huPrP(23−111) was adjusted to 25 ml with digestion buffer and the cells were disrupted with a VS 70 T sonotrode, 70% amplitude, 3 s pulse, 5 s pause for 2 × 5 min on ice with a 5-min break. The lysate was centrifuged at 28,700 × *g* and 4 °C for 1 h. After confirmation that huPrP(23−111) is located in the insoluble inclusion bodies, the pellet was dissolved in about 10 ml of 6 M guanidinium HCl, 30 mM Tris-HCl, pH 7.4 at 4 °C overnight and centrifuged again. 25 mM imidazole was added to the supernatant, which was used for immobilized metal ion affinity chromatography using a 5 ml Protino nickel-nitrilotriacetic acid column. The elution of the hexahistidine-tagged huPrP(23−111) occurred with a linear gradient of 75 ml from 25 to 500 mM imidazole in 6 M guanidinium HCl, 30 mM Tris-HCl, pH 7.4. huPrP(23−111) containing fractions were purified by RP-HPLC. A semipreparative C8 column (Zorbax 300 SB-C8, 9.4 × 250 mm (Agilent)) allowed the purification of huPrP(23−111) from impurities and salts within the buffer (especially guanidinium HCl and imidazole) using a 12−24% (v/v) gradient of acetonitrile + 0.1% (v/v) TFA in Milli-Q water within 20 min. The purification was performed at 80 °C at a flow rate of 4 ml min^−1^. The elution fractions containing huPrP(23−111) were pooled and dried by lyophilization. For removal of the N-terminal hexahistidine tag, the lyophilizate was diluted in 8 ml of 50 mM Tris-HCl, pH 7.4 and 3.8 mg TEV protease was added to the protein for 5−10 days at 4 °C. The digested huPrP(23−111) was purified by RP-HPLC (see above) and huPrP(23−111) containing fractions were lyophilized. The protein was dissolved in Milli-Q water to a final concentration of 253 µM, flash-frozen in liquid N_2_ and stored at −80 °C.

The fragments huPrP(23−111Δ41−94), huPrP(23−40) and huPrP(95−111) were obtained as synthetic peptides from either peptides&elephants or Caslo, respectively. The peptides were dissolved in Milli-Q water to a stock concentration of ~250 µM which was confirmed by photometric measurements.

### Purification of αSyn

αSyn in the pT7-7 vector was expressed in *E. coli* BL21 (DE3). To facilitate N-terminal acetylation in αSyn, the N-terminal acetylation enzyme NatB from *Schizosaccharomyces pombe* was coexpressed in a second vector, pNatB^43^. Expression was conducted in 50 mM phosphate-buffered 2YT-medium (pH 7.2) with 0.4% glycerol and 2 mM MgCl_2_, protein production was induced at OD_600_ 1−1.2 with 1 mM isopropyl *β*-d-1-thiogalactopyranoside and ran for 4 h at 37 °C. Purification of acetylated αSyn was carried out as previously described^44^. After ion exchange chromatography, αSyn was further purified by RP-HPLC on a Zorbax 300 SB-C8, 9.4 × 250 mm column (Agilent) using a gradient from 30 to 40% acetonitrile, 0.1% TFA in Milli-Q water, run over 20 min. The peak corresponding to αSyn was collected, flash-frozen in liquid N_2_ and lyophilized.

ATTO633 maleimide labeling of αSyn A140C was performed as follows: αSyn A140C was prepared as described^45^, reduced by addition of 25 mM TCEP and incubated for 1 h at RT. Reduced, monomeric αSyn A140C was then purified by RP-HPLC as described before. After lyophilization, 3 mg αSyn A140C was dissolved in 1 ml of 200 mM sodium phosphate buffer, pH 7.4, already containing a twofold molar excess (~0.3 mg) of ATTO633-maleimide predissolved in 60 µl *N*,*N*-dimethylformamide. Labeling was performed at RT for 2 h and 600 rpm agitation on a microcentrifuge tube shaker. Labeled αSyn A140C-ATTO633 was separated from free label by RP-HPLC. After 2 min at 30% acetonitrile + 0.1% TFA in Milli-Q water, a gradient from 30 to 40% acetonitrile + 0.1% TFA in Milli-Q water was run within 20 min at 4 ml min^−1^, the labeled protein peak was collected and lyophilized.

### Preparation of αSynO

The preparation of αSynO is based on the protocols of Giehm et al.^32^ and Lorenzen et al.^33^. Purified αSyn was dialyzed in a Slide-A-Lyzer MINI dialysis device (3.5 kDa MWCO, Thermo Scientific) against Milli-Q water either overnight at 4 °C or for 2 h at room temperature. The dialyzed protein was transferred to LoBind reaction tubes (Eppendorf AG), flash-frozen with liquid N_2_ and lyophilized or dried in a rotational vacuum concentrator system connected to a cold trap (both Martin Christ Gefriertrocknungsanlagen GmbH). The lyophilizates were dissolved at 12 mg ml^−1^ in 30 mM Tris, 50 mM NaCl, pH 7.4, and incubated at 37 °C, 900 rpm for 3−5 h. Subsequently, the solution was centrifuged at 16,100 × *g* for 10 min and the supernatant was loaded onto an SEC column (Superdex 200 Increase 10/300 GL, GE Healthcare). The SEC was performed in 30 mM Tris, 50 mM NaCl, pH 7.4, at room temperature and a flow rate of 0.75 ml min^−1^. αSynO containing fractions were united and concentrated (Vivaspin 500, 3 kDa MWCO, Sartorius) to typically 30−110 µM (monomer concentration) and stored at 4 °C.

### Density gradient ultracentrifugation

*Sample preparation*: 10 µM of αSynO or αSyn mono were coincubated with 2−20 µM of huPrP(23−144), or 20 µM of either huPrP(23−230), huPrP(23−111), huPrP(90−230), huPrP(121−230), huPrP(23−111Δ41−94), huPrP(95−111) or huPrP(23−40) in 30 mM Tris-HCl, pH 7.4 for 1.5 h at room temperature. The final volume of each sample was 100 µl. For preparation of mixtures of αSynO, AβO and huPrP (huPrP(23−230), huPrP(23−144) or huPrP(23−111)), 80 µM of Aβ(1−42) (obtained from Bachem; for preparation of Aβ(1−42) stocks, see ref. ^26^) was incubated for 2 h at 22 °C and 600 rpm shaking in 30 mM Tris-HCl, pH 7.4 to obtain AβO. 40 µM AβO and 10 µM or 40 µM αSynO were united before either 2, 4 or 5 µM of huPrP was added for further 30 min. The final volume of each sample was 100 µl. As controls, 40 µM AβO was analyzed alone or with 10 µM αSynO.

*DGC*: The method used is based on the QIAD protocol^35^. Density gradient ultracentrifugation was performed as previously described^26^. In short, each sample (100 µl) was applied onto a discontinuous 30 mM Tris-HCl, pH 7.4 buffered sucrose gradient layered in an 11 mm × 34 mm centrifuge tube. The gradients were centrifuged for 3 h at 259,000 × *g* and 4 °C in an Optima MAX-XP ultracentrifuge (Beckman Coulter) using a TLS-55 swing-out rotor (Beckman Coulter) and manually fractionated into 13 142-µl fractions. The last fraction (14) was formed by addition of 80 µl of 30 mM Tris-HCl, pH 7.4 buffer to the remaining volume.

### SDS-polyacrylamide gel electrophoresis and silver staining

Density gradient ultracentrifugation fractions were analyzed qualitatively by SDS-PAGE and silver staining. Therefore, each fraction was diluted 1:1 in sample buffer (12% glycerol, 4% SDS, 50 mM Tris-HCl, pH 7.4, 2% *β*-mercaptoethanol) and 15 µl of each fraction was applied onto 15% Tris/Glycine gels containing a 7% stacking gel prepared according to standard protocols. Electrophoresis was performed at a constant voltage of 130 or 140 V. Proteins were visualized by silver staining of the gels based on the protocol by Heukeshoven and Dernick^46^.

In case of the sample “10 µM αSynO + 20 µM huPrP(95−111)”, SDS-PAGE was performed on a 20% Tris/Tricin gel containing a 5.6% stacking gel as described previously^26^.

### RP-HPLC analysis

For quantitative analysis of Aβ, αSyn, and huPrP and determination of αSyn:huPrP ratios within formed heteroassemblies (DGC fractions 11−14), RP-HPLC was performed as described previously^26^. In short, 20 µl of the DGC fractions was applied on a Zorbax 300 SB-C8 Stable Bond Analytical column, 4.6 × 250 mm (Agilent) and measured with an Agilent 1260 infinity system. A gradient from 10 to 40% (v/v) acetonitrile + 0.1% (v/v) TFA within 25 min at 80 °C and a flow rate of 1 ml min^−1^ allowed the separation of each protein. Histograms were plotted with OriginPro 9.0G.

### Dynamic light scattering

Heteroassemblies derived from 10 µM αSynO and 20 µM huPrP(23−144) were prepared by pooling sucrose DGC fractions 12 and 13 of two samples to receive enough volume for the measurement. Dynamic light scattering was performed on a submicron particle sizer, Nicomp 380 (Particle Sizing Systems Nicomp, Santa Barbara, CA). Data were analyzed with the Nicomp algorithm using the volume-weighted Nicomp distribution analysis. For data analysis, a measured refractive index in the sample of 1.431 corresponding to 54.5% sucrose and a viscosity of 26 centipoise was taken into account^47^. For heteroassemblies derived from 10 µM αSynO and 20 µM huPrP(23−111) or from 10 µM αSynO and 20 µM huPrP(23−230), DGC fractions 12−14 were pooled. In case of the huPrP(23−111) sample, a refractive index of 1.4125 (46.5% sucrose) and a viscosity of 10 centipoise were used. For αSynO−huPrP(23−230) heteroassemblies, a refractive index of 1.4085 (44.25% sucrose) and a viscosity of 8.6 centipoise were taken into account.

### Atomic force microscopy

For sample preparation, 10 μM (monomer concentration) αSynO (containing ~5 mM NaCl) was incubated for 1 h at room temperature alone or with 2, 5 or 10 μM huPrP(23−144), huPrP(23−230), or huPrP(23−111) in 30 mM Tris-HCl, pH 7.4 in LoBind reaction tubes (Eppendorf AG). Next, 5 μl of each sample was put onto a freshly cleaved muscovite mica surface and incubated for 10 min under humid atmosphere to avoid drying, followed by washing with Milli-Q water (100 μl, three times) and drying with N_2_ gas. Imaging was performed in intermittent contact mode (AC mode) in a JPK Nano Wizard 3 atomic force microscope using a silicon cantilever with silicon tip (OMCL-AC160TS-R3, Olympus) with a typical tip radius of 9 ± 2 nm, a force constant of 26 N m^−1^ and a resonance frequency around 300 kHz. The images were processed using JPK Data Processing Software (version spm-5.0.84). For the presented height profiles, a polynomial fit was subtracted from each scan line first independently and then using limited data range. Moreover, in order to improve the visual representation of the substructures of the complexes, we additionally performed edge detection using the Sobel operator in both *X* and *Y* directions for each height profile correspondingly. Several AFM images were recorded for every condition and representative images are shown.

### Total internal reflection fluorescence microscopy

Fluorescently labeled AβO was prepared by mixing synthetic Aβ(1−42) with 10% (mol/mol) FITC-Aβ(1−42) with an N-terminal FITC label (both from Bachem) and preincubated as described^26^. Fluorescently labeled αSynO was prepared by applying the oligomer preparation protocol as described above, with 10% (mol/mol) αSyn A140C with a C-terminal ATTO633 label (ATTO-TEC) present during lyophilization of a 6 mg oligomer batch.

For TIRF microscopy of AlexaFluor488-labeled huPrP(23−144), 2 µM of huPrP (23−144) was mixed with 0.2 µM huPrP(23−144)-Cys-AlexaFluor488 and 10 µM αSynO in 10 µl. For coclustering of αSynO and AβO, 2 µM of huPrP(23−144) was mixed with 10 µM ATTO633-labeled αSynO and/or 40 µM of preincubated FITC-labeled AβO in 10 µl. Seven microliters of these solutions was deposited onto cleaned glass slides (Coverslips #1, 0.13−0.16 mm thickness, 25 × 60 mm, Menzel-Gläser) and dried at RT. TIRF microscopy was performed as described^48^. For excitation of FITC-labeled AβO, a 488 nm laser in combination with a 525 nm bandpass filter was used. For excitation of ATTO633-labeled αSynO, a 635 nm laser with a 705 nm bandpass filter was used.

### Biolayer interferometry

huPrP(23−144)-Cys-biotinyl was attached to streptavidin (SA) biosensors (fortéBIO, PALL Life Science) via streptavidin−biotin coupling and either αSyn mono or αSynO were used as analyte. Before usage, SA biosensors were hydrated in 30 mM Tris-HCl, pH 7.4. Binding of αSyn mono and αSynO was compared on a BLItz system (fortéBIO, PALL Life Science). Association was recorded for 600 s for 2 µM of either αSyn mono or αSynO in 30 mM Tris-HCl, 50 mM NaCl, pH 7.4 followed by a dissociation step for further 600 s in 30 mM Tris-HCl, 50 mM NaCl, pH 7.4. The same buffer was used as reference. Concentration-dependent binding of αSynO was analyzed on an Octet RED96 instrument (fortéBIO, PALL Life Science). huPrP(23−144)-Cys-biotinyl-coated biosensors were additionally quenched with 100 µM biotin before measurement. A dilution series of αSynO from 250 to 16 nM diluted in 30 mM Tris-HCl, 50 mM NaCl, pH 7.4 was recorded for 600 s (association) followed by a dissociation step of further 1200 s. Furthermore, biotin-coated biosensors were used as reference. The sensorgrams were double referenced using the biotin-coated biosensors and a sample containing only 30 mM Tris-HCl, 50 mM NaCl, pH 7.4 buffer. Curves were plotted with OriginPro 9.0G.

### CD spectroscopy

8 µM huPrP(23−144) or αSynO (containing ~5 mM NaCl) in 10 mM Tris-HCl, pH 7.4, were transferred into a cuvette (110-QS, 1 mm, Hellma Analytics) and analyzed by CD spectroscopy. Spectra were recorded from 195 to 260 nm at 20 °C and a scan speed of 100 nm min^−1^ in a Jasco J-815 spectropolarimeter. Subsequently, huPrP(23−144) concentrations from 1 to 8 µM were titrated to the 8 µM αSynO sample and spectra were recorded after each titration step. Spectra were smoothed by averaging the CD signal over the range wavelength ±1 nm.

### Solution NMR spectroscopy

αSynO was gradually added to a sample of [U-^13^C,^15^N] huPrP(23−144) (initial concentration 60 µM) in 30 mM Tris-HCl, pH 7.4, 10% (v/v) D_2_O. Two-dimensional [^1^H,^15^N] HSQC NMR spectra^49^ were recorded at 5.0 °C on a Bruker AVANCE NEO 900 MHz NMR spectrometer equipped with a cryogenically cooled triple resonance probe with *z*-axis pulsed field gradient capabilities. The sample temperature was calibrated using methanol-d_4_ (99.8%)^50^. The ^1^H_2_O resonance was suppressed by gradient coherence selection, with quadrature detection in the indirect ^15^N dimension achieved by the echo-antiecho method^51,52^. A WALTZ-16 sequence^53^ with a field strength of 1.3 kHz was employed for ^15^N decoupling during acquisition. 1280 (256) complex data points were acquired with a spectral width of 16 p.p.m. (26.0 p.p.m.) in the ^1^H (^15^N) dimension. All NMR spectra were processed using NMRPipe and NMRDraw^54^ and analyzed with NMRViewJ^55^. ^1^H chemical shifts were referenced with respect to external DSS in D_2_O and ^15^N chemical shifts were referenced indirectly^56^. A median baseline correction algorithm^57^ was used in the direct dimension to remove any baseline offsets. To quantify the total amide signal intensity, all data points in the 2D [^1^H,^15^N] HSQC NMR spectra in the backbone amide proton region from 7.95 to 8.70 p.p.m. and in the tryptophan indole proton region from 10.00 to 10.20 p.p.m. were integrated using NMRPipe^54^. The resulting amide signal intensity was corrected for sample dilution along the titration, number of transients collected, and sensitivity of the ^1^H transmitter/receiver coil (which is inversely proportional to the calibrated ^1^H pulse length) as appropriate^58^.

### Statistics and reproducibility

Density gradient ultracentrifugation experiments were typically performed three times (*n* = 3) per construct/condition and showed full consistency regarding the main outcomes (i.e., HMW heteroassociates formed yes/no; monomer/oligomer fraction decreased/disappeared yes/no). For the construct/condition in Fig. 2h, *n* = 2. For the constructs/conditions in Figs. 2g, j–l and 7a–d, *n* = 1. The huPrP−αSynO interaction was furthermore confirmed by several complementary techniques, which again were repeated (e.g., fluorescence microscopy with either huPrP or αSynO/AβO labeled; biolayer interferometry on two different instruments). All attempts to replicate the data were successful.

### Reporting summary

Further information on research design is available in the Nature Research Reporting Summary linked to this article.

## Supplementary information

Supplementary Information

Supplementary Data 1

Reporting Summary

## Data Availability

The source data underlying Figs. 3b, 4l, 5, 6, 7e, f are provided in Supplementary Data 1. Other relevant data are available from the corresponding author upon request.
