# Supplementary material for: Clustering of human prion protein and α-synuclein oligomers requires the prion protein N-terminus
Source: Commun Biol. 2020 Jul 9;3:365. doi: 10.1038/s42003-020-1085-z (PMC7347944; doi:10.1038/s42003-020-1085-z)
Supplement: Supplementary file 1 — Supplementary Information [file 42003_2020_1085_MOESM1_ESM.pdf]

Supplementary Information

Clustering of human prion protein and α-synuclein oligomers requires the prion protein N-terminus

Nadine S. Rösener[1,2], Lothar Gremer[1,2], Michael M. Wördehoff[1], Tatsiana Kupreichyk[1,2], Manuel Etzkorn[1,2], Philipp Neudecker[1,2], and Wolfgang Hoyer[1,2,*]

[1]Institut für Physikalische Biologie, Heinrich-Heine-Universität Düsseldorf, 40204 Düsseldorf, Germany

[2]Institute of Biological Information Processing (IBI-7) and JuStruct: Jülich Center for Structural Biology, Forschungszentrum Jülich, 52425 Jülich, Germany

Correspondence and requests for materials should be addressed to W.H. (email: wolfgang.hoyer@hhu.de)

Supplementary Figures

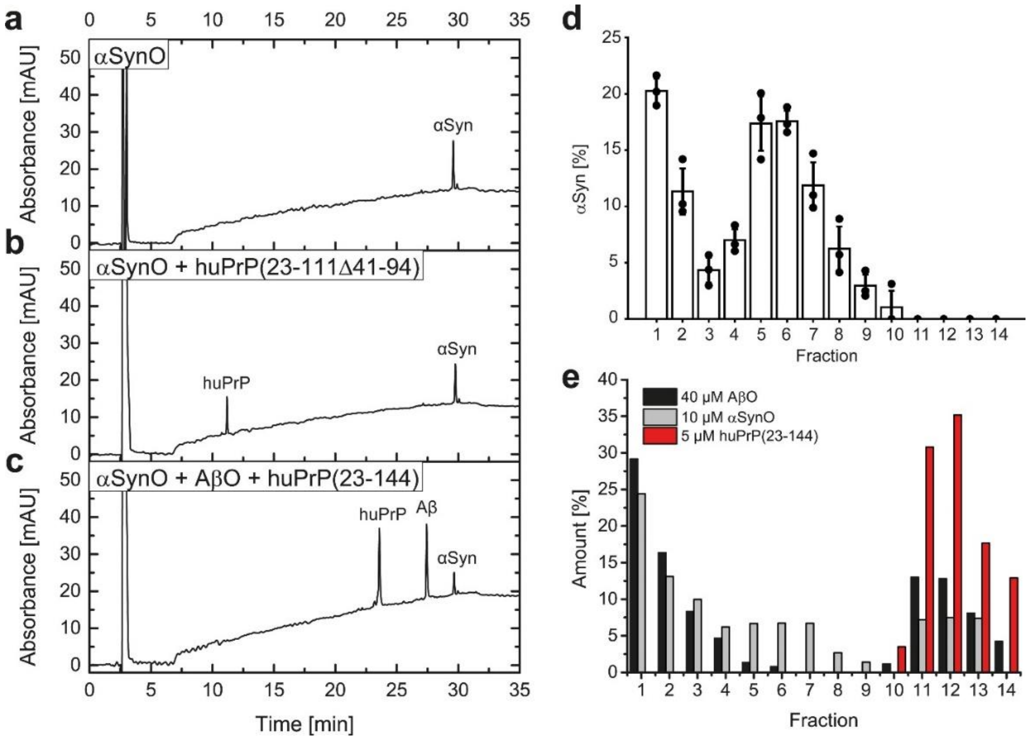

Supplementary Fig. 1 Quantification of protein contents of DGC fractions by RP-HPLC. The individual DGC fractions were analyzed to determine the αSyn, Aβ, and huPrP contents. **a** Example chromatogram of one DGC fraction of an αSynO sample. **b** Chromatogram of a DGC HMW fraction showing that huPrP(23-111Δ41-94) co-migrates with αSyn. **c** Example chromatogram of one DGC HMW fraction of a mixture of αSynO, AβO, and huPrP(23-144). **d** Determination of the monomer content in αSynO samples. Plot of the distribution of αSyn over the 14 fractions after DGC of αSynO samples. The bars represent mean and standard deviation of three independent αSynO preparations. The residual monomer content (fractions 1-3) accounts for 35.9 ± 3.2 % of total αSyn (error represents SD). **e** Plot of the distribution of αSyn, Aβ, and huPrP(23-144) over the 14 fractions after DGC of a sample containing 10 µM αSynO, 40 µM AβO, and 5 µM huPrP(23-144).

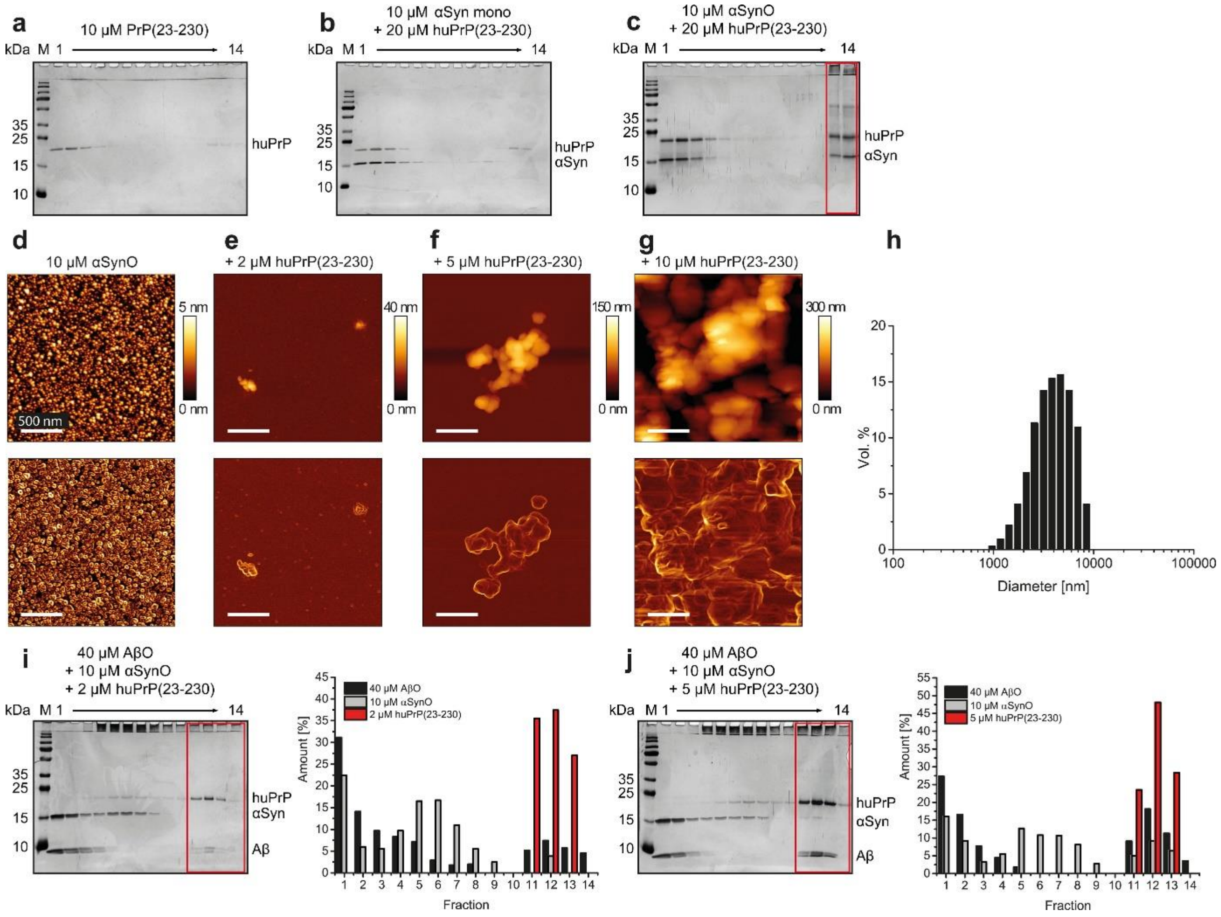

Supplementary Fig. 2 Characterization of cluster formation of αSynO and huPrP(23-230) and co-cluster formation with AβO. **a-c** Silver stained Tris/Glycine SDS-PAGE gels show the distributions of the applied proteins within the DGC gradients from left to right corresponding to the fractions from top to bottom of each gradient. Lanes corresponding to HMW heteroassemblies are marked by red boxes. **d-g** AFM of αSynO **d** without and **e-g** with increasing concentrations of huPrP(23-230). AFM data are shown both as raw height images (top panels) and after edge detection using the Sobel operator for visualization of structural details (bottom panels). AFM scale bars represent 500 nm. **h** DLS measurement of heteroassemblies generated from 10 µM αSynO and 20 µM huPrP(23-230) and isolated by sucrose DGC. **i**, **j** αSynO and AβO co-cluster with huPrP(23-230). Silver stained SDS-PAGE gels and quantitative analysis by RP-HPLC after DGC of mixtures of 40 µM AβO and 10 µM αSynO containing either **i** 2 µM or **j** 5 µM huPrP(23-230). Co-clustering of all proteins as heteroassemblies is detectable in DGC fractions 11-14 (red boxes).

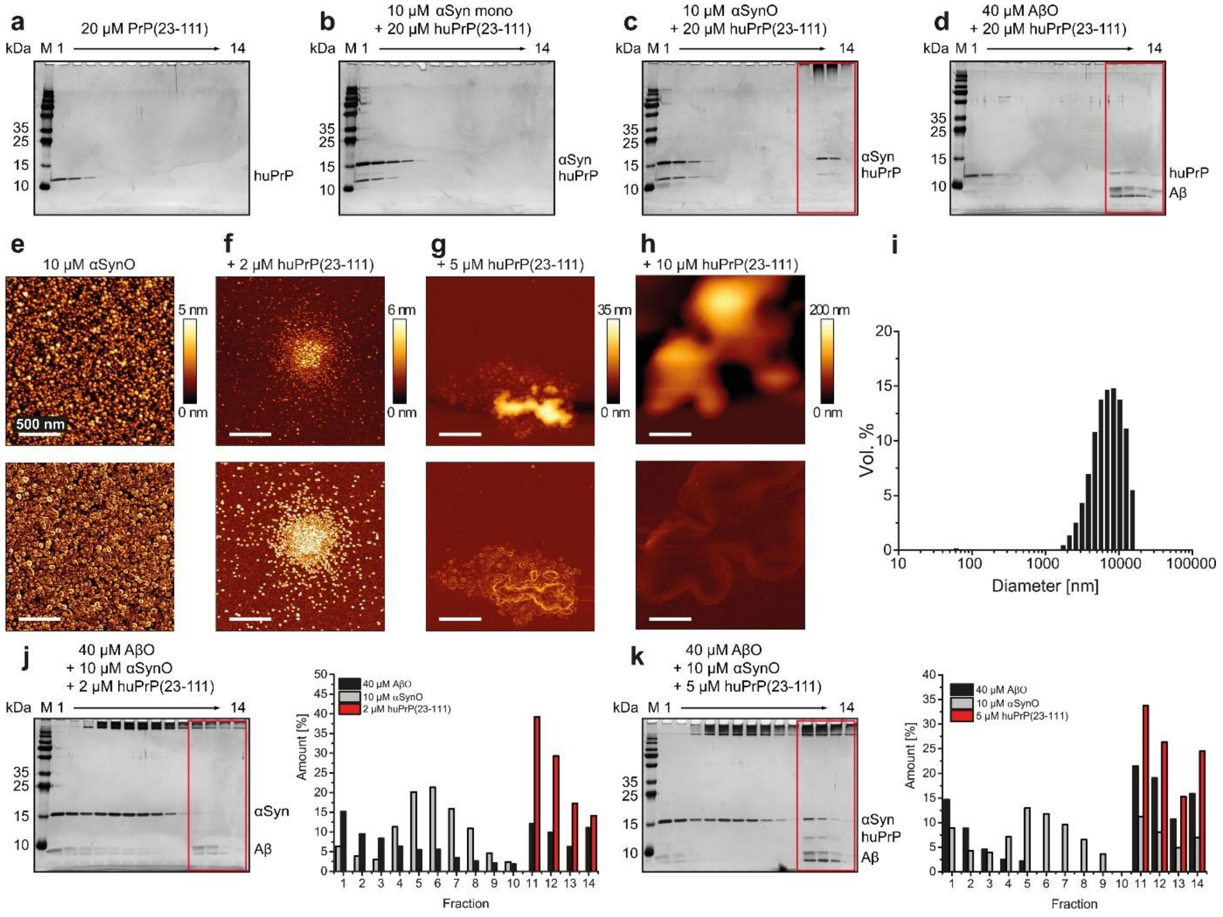

Supplementary Fig. 3 Characterization of cluster formation of αSynO and huPrP(23-111) and co-cluster formation with AβO. **a-d** Silver stained Tris/Glycine SDS-PAGE gels show the distributions of the applied proteins within the DGC gradients from left to right corresponding to the fractions from top to bottom of each gradient. Lanes corresponding to HMW heteroassemblies are marked by red boxes. **e-h** AFM of αSynO **e** without and **f-h** with increasing concentrations of huPrP(23-111). AFM data are shown both as raw height images (top panels) and after edge detection using the Sobel operator for visualization of structural details (bottom panels). AFM scale bars represent 500 nm. **i** DLS measurement of heteroassemblies generated from 10 µM αSynO and 20 µM huPrP(23-111) and isolated by sucrose DGC. **j**, **k** αSynO and AβO co-cluster with huPrP(23-111). Silver stained SDS-PAGE gels and quantitative analysis by RP-HPLC after DGC of mixtures of 40 µM AβO and 10 µM αSynO containing either **j** 2 µM or **k** 5 µM huPrP(23-111). Co-clustering of all proteins as heteroassemblies is detectable in DGC fractions 11-14 (red boxes).

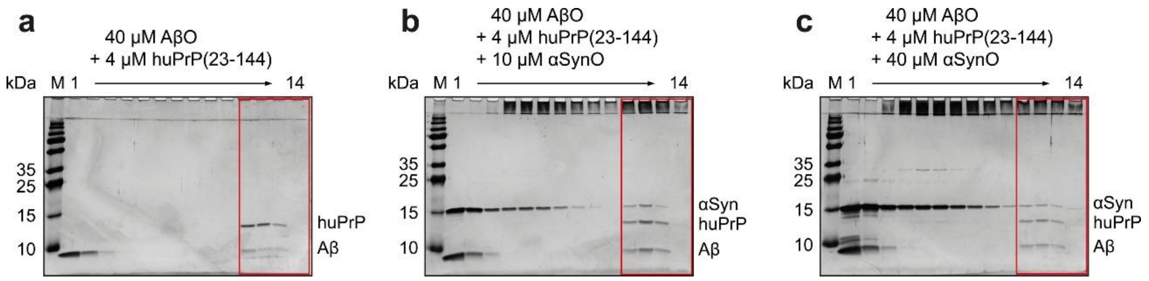

Supplementary Fig. 4 αSynO and AβO compete for cluster formation with huPrP(23-144). Silver stained SDS-PAGE gels after DGC of a mixture of 4 μM huPrP(23-144) and 40 μM AβO containing either **a** no, **b** 10 μM, or **c** 40 μM αSynO. The quantitative analysis by RP-HPLC of the distribution of assemblies into oligomer fractions (4-9) vs. HMW fractions (10-14) is shown in Fig. 7f.

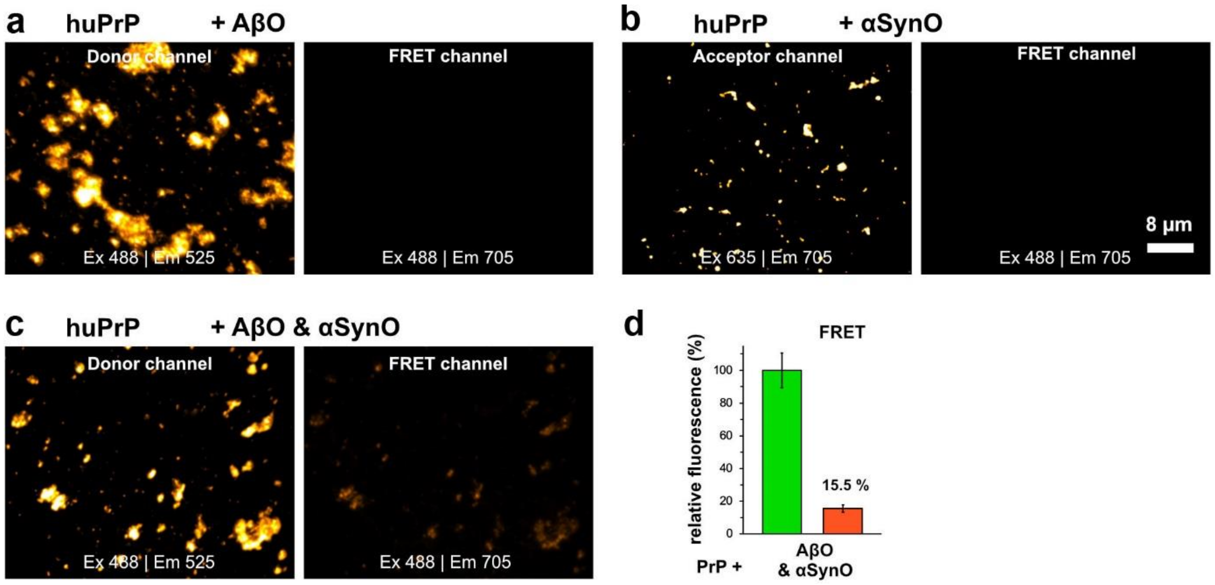

Supplementary Fig. 5 FRET between AβO and αSynO in mixed condensates with huPrP(23-144) observed by TIRFM. The images are the same as those presented in Fig. 7. AβO was 10 % FITC-labeled, αSynO was 10 % Atto633-labeled, huPrP(23-144) was not fluorescently labeled. **a** 2 µM huPrP(23-144) and 40 µM AβO, excited at 488 nm and detected at 525 nm (donor channel, left) and the corresponding image with excitation at 488 nm and detection at 705 nm (FRET channel, right). **b** 2 µM huPrP(23-144) and 10 µM αSynO, excited at 635 nm and detected at 705 nm (acceptor channel, left) and the corresponding image with excitation at 488 nm and detection at 705 nm (FRET channel, right). **c** Mixture of 2 µM huPrP(23-144), 40 µM AβO and 10 µM αSynO, excited at 488 nm and detected at 525 nm (donor channel, left) and the corresponding image with excitation at 488 nm and detection at 705 nm (FRET channel, right). Only the mixed condensates **c** show a fluorescence signal in the FRET channel. **d** Relative fluorescence intensity in the FRET channel of mixed huPrP, AβO and αSynO condensates (red bar) compared to the donor channel (set to 100 %, green bar). Background fluorescence of the controls (huPrP with AβO and αSynO alone) was subtracted. Thirty two 40 µm x 50 µm areas as shown in **c** were evaluated for the bar chart. The mean of their integrated density (=sum of pixel intensities) was calculated with ImageJ (developed by Wayne Rasband, National Institutes of Health, Bethesda, MD). Error bars represent SD among the 32 evaluated areas. -- Scale bar for all images: 8 µm.

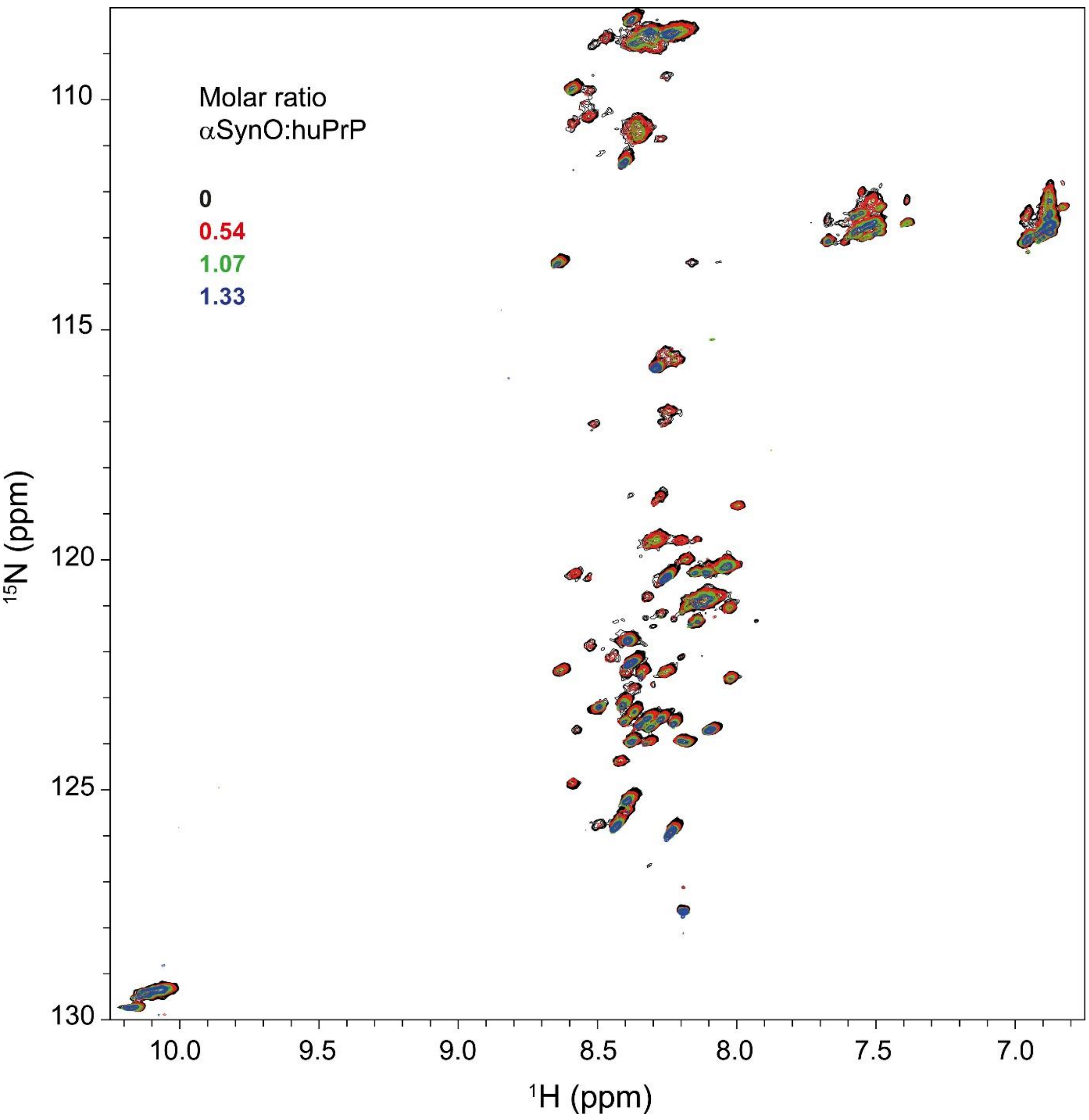

Supplementary Fig. 6 [^{1}H,^{15}N] HSQC NMR spectra of [U-^{13}C,^{15}N]-labeled huPrP(23-144) before (black) and after addition of unlabeled αSynO to the indicated molar ratio. αSynO was gradually added to a sample of [U-^{13}C,^{15}N] huPrP(23-144) (initial concentration 60 µM) in 30 mM Tris-HCl, pH 7.4, 10 % (v/v) D_2O. This figure is an enlarged copy of Fig. 3a.
